# TPH1 gene polymorphism rs211105 is associated with serotonin and tryptophan hydroxylase 1 concentrations in acute pancreatitis patients

**DOI:** 10.1186/s12876-021-02012-z

**Published:** 2021-11-12

**Authors:** Jadwiga Snarska, Ewa Fiedorowicz, Dominika Rozmus, Konrad Wroński, Maria Latacz, Natalia Kordulewska, Janusz Płomiński, Roman Grzybowski, Huub F.J. Savelkoul, Elżbieta Kostyra, Anna Cieślińska

**Affiliations:** 1 Department of General Surgery, Faculty of Medical Sciences, University of Warmia and Mazury, Olsztyn, Poland; 2 Department of Biochemistry, Faculty of Biology and Biotechnology, University of Warmia and Mazury in Olsztyn, Olsztyn, Poland; 3 General and Colorectal Surgery Clinic, University Clinical Hospital of the Military Medical Academy - Central Veterans Hospital in Lodz, Lodz, Poland; 4 Department and Clinic of Orthopaedics and Traumatology, Collegium Medicum, University of Warmia and Mazury, 10-719 Olsztyn, Poland; 5 Cell Biology and Immunology Group, Department of Animal Sciences, Wageningen University and Research, Wageningen, Netherlands

**Keywords:** AP, Polymorphism, Acute pancreatitis, rs211105, TPH1, Tryptophan hydroxylase 1

## Abstract

**Background:**

The role of serotonin and its metabolic pathway in proper functioning of the pancreas has not been thoroughly investigated yet in acute pancreatitis (AP) patients. Tryptophan hydroxylase (TPH) as the rate-limiting enzyme of serotonin synthesis has been considered for possible associations in various diseases. Single-nucleotide polymorphisms (SNPs) in TPH genes have been already described in associations with psychiatric and digestive system disorders. This study aimed to explore the association of a rs211105 (T/G) polymorphism in TPH1 gene with tryptophan hydroxylase 1 concentrations in blood serum in a population of acute pancreatitis patients, and to investigate this association with acute pancreatitis susceptibility.

**Results:**

Our data showed an association between the presence of the T allele at the position rs211105 (OR = 2.47, 95 % CI 0.94–6.50, p = 0.06) under conditions of a decreased AP incidence. For TT and GT genotypes in the control group, the lowest concentration of TPH was associated with higher serotonin levels (TT: Rs = − 0.415, p = 0.0018; GT: Rs = − 0.457, p = 0.0066), while for the AP group the highest levels of TPH among the TT genotype were associated with lower levels of serotonin (TT: Rs = − 0.749, p < 0.0001, and in the GG genotype higher levels of TPH were associated with higher levels of serotonin (GG: Rs = − 0.738, p = 0.037).

**Conclusions:**

Here, a new insight in the potential role of a selected genetic factor in pancreatitis development was shown. Not only the metabolic pathway of serotonin, but also factors affecting serotonin synthesis may be interesting and important points in acute pancreatitis.

## Introduction

Ischaemia, bile duct obstruction, activation of pancreatic protease as well as pro-inflammatory cytokines are important components in the etiopathogenesis of acute pancreatitis (AP) [1, 2]. AP may have an unpredictable course. Therefore, there is an urgent need for determination of the prognostic symptoms that would enable to identify patients at high risk of severe course [3, 4]. Till now, more severe AP has been associated with older age, obesity, pancreatic necrosis, fluid collection, organ failure and some genetic factors [5]. The role of serotonin and its metabolic pathway in the proper functioning of the pancreas has not been thoroughly investigated yet with respect to AP.

Serotonin (5-HT, 5-hydroxytryptamine) is a monoamine neurotransmitter, synthesized in serotonergic neurons of the central nervous system (CNS), in enterochromaffin cells (EC) present in the gastrointestinal epithelium [6] and also, immune system cells as macrophages, and T cells [7]. However, it is known that the brain-derived serotonin provides only about 5 % of total body serotonin, while 95 % of serotonin is produced in the peripheral organs, mostly in the gut. These findings triggered further research on serotonin function in multiple physiology aspects [8].

**Fig. 1:**
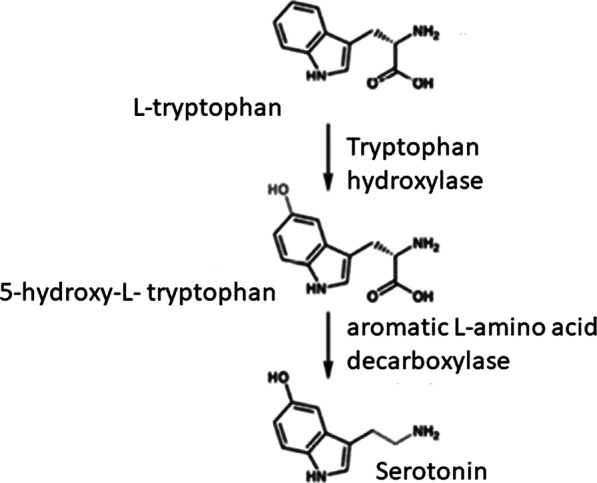
Scheme of serotonin synthesis from L-tryptophane (based on El-Merahbi et al. [8] modified)

The 2-step enzymatic synthesis pathway starts from dietary L-tryptophan conversion into 5-hydroxy-L-tryptophan (5-HTP) and then to 5-HT by the activity of two enzymes: tryptophan hydroxylase (TPH) and ubiquitous aromatic L-amino acid decarboxylase (AADC), respectively (Fig. 1). 5-HT is degraded into 5-hydrosyindoleacetic acid by monoamine oxidase A (5-HIAA) [7, 9].

Serotonin regulates emotional expression and social behaviour, but also proliferation of immune system cells, muscle and epithelial cells, and neurons [6, 10, 11]. It is also a chemotactic molecule for such cells as eosinophils, dendritic cells and mast cells [7]. It has been proven that the intracellular content of serotonin correlates positively with the insulin secretion rate, and TPH1-deficient mice showed the development of a mild form of diabetes as a result of impaired insulin secretion in the pancreas [12]. Almaca et al. [13] found that serotonin is a paracrine signal released by human pancreatic β-cells and regulating glucagon secretion. The effect of serotonin deficiency, the lack of its transporters, receptors or enzymes of the serotonin pathway are implicated in many diseases, including depression, numerous mood swings, emotional instability, schizophrenia and other neurological disorders, and irritable bowel syndrome [14–22].

Because the serotonin concentration is regulated by the rate-limiting enzyme TPH, this TPH has been considered for possible associations with suicidal behaviour [19, 20, 23, 24], irritable bowel syndrome [18], or depression [25]. Presently, two forms of TPH were identified (TPH1 and TPH2), with TPH2 being expressed mainly in the brain, while TPH1 is expressed in brain and EC cells in the gut [18].

The human TPH1 gene has been cloned and mapped to chromosome 11p15.3-14 (Gene ID: 7166); it has 11 exons [19, 24] and consists of 444 amino acids [18].Single-nucleotide polymorphisms (SNPs) have been described in genes coding for type one and type two tryptophan hydroxylases in association with psychiatric disorders and suicidal behaviour [26–29]. There are also a few studies investigating the role of the rs211105 polymorphism in diarrhoea-predominant irritable bowel syndrome, and the digestive system [18, 21, 22]. However, to the best of our knowledge, there are no published data on TPH1 polymorphism rs211105 in correlation to serotonin hydroxylase 1 activity or serotonin concentrations in blood serum.

In the present study, we investigated the association of the TPH1 gene polymorphism rs211105 (T/G) with acute pancreatitis and tryptophan hydroxylase 1 concentrations in circulation. Due to the fact that tryptophan hydroxylase 1 is also expressed in β-cells of the pancreas [6], we predict its role in proper pancreas functioning.

## Materials and methods

### Ethics and general information

Specialists recruited all 198 participants either at the Department of General Surgery and Oncology of the Warmia and Mazury University Hospital in Olsztyn or at the Clinical Department of Trauma-Orthopedic Surgery and Spine Surgery of the Provincial Specialist Hospital in Olsztyn in 2014-2020. All participants were treated according to the Patient Right Protection Act of our institution and international guidelines, and the Local Bioethics Committee approved our study (13/2016; 51/2019).

Peripheral blood samples (5-10 ml) was collected into a tube containing EDTA, were collected from each patient by the medical staff, and all biological material was immediately transported to the laboratory and directly used in analysis or stored at -80 °C.

### Controls and AP group characteristic

Our study included 198 individuals (all Caucasian): 107 patients diagnosed with AP (19 females and 88 males; mean age ranging from 28 to 76 years; average 52.4) and 91 healthy people (25 females and 66 males; mean age ranging from 23 to 68 years; average 44.2). This is therefore a cross-sectional study.

Patients were admitted to the hospital 8-36 h after the onset of AP symptoms (pain, vomiting, emetic reflex). Comorbidity of chronic circulatory system, liver, kidney or lung diseases caused exclusions from the study. Blood samples were collected from the forearm vein for the panel of biochemical tests twice – upon arrival at the hospital and 48 h after admission. Within 2 days, each patient had computed tomography (CT) with contrast performed to detect fluid collections, the extent of inflammation or necrotic changes. APACHE-II (Acute Physiology And Chronic Health Evaluation II) scores were calculated using data from the first 24 h after admission to assess patients’ condition. Predicting acute pancreatitis severity and potential complications were based on imaging scales performed 3-4 days after the onset of symptoms, then after 10-12 days treatment.

For AP group, primarily patients with alcohol-related acute pancreatitis were selected for the study. 75 % of AP cases met the DSM IV criteria for alcohol dependence, and 25 % - incidental cause of inflammation. Both groups presented no inflammatory disease or other infection symptoms, no urogenital tract or kidney failure. This was confirmed by laboratory tests, and all required data was collected from their medical records and/or a filled-in questionnaire. The control group included individuals after control visit at hospital, and volunteers. Both groups were matched in age, and gender ratio. Table 1 presents the characteristics of both groups.

**Table 1:** Distribution of selected characteristics in acute pancreatitis patients and healthy Control group

Characteristic	Controls	AP	*p*
	n = 91	n = 107	
Age (years)	44.2 (±10.2)	52.4 (±13.3)	0.5
Body mass (kg)	70.1 (±12.3)	75.6 (±11.8)	0.6
Amylase activity in serum (IU/L)	79.2. (±24.7)	1647.5 (±636.5)	< 0.001
Lipase (IU/L)	114.7 (±40.4)	1446.7 (±814.6)	< 0.001
Bilirubin (mg/dL)	0.6 (±0.21)	1.9 (±0.87)	0.05
Glucose (mg/dL)	90.2 (±8.4.)	127.4 (±33.9)	0.05
AST (IU/L)	37.2 (±6.4)	155.2 (±71.6)	< 0.001
ALT (IU/L)	26.2 (±2.1)	155.7 (±30.2)	< 0.001
CRP (mg/dL)	0.31 (±0.6)	4.4 (±1.4)	< 0.001
Female (%)	27.4	17.7	
APACHE II scale	N.a.	4.4 (±1.4)	

p-values were determined by Tukey’s test

### Polymorphism rs211105 in TPH1 gene in healthy and AP patients

DNA was isolated from peripheral blood using GeneJET™ Whole Blood Genomic DNA Purification Mini Kit (Thermo Scientific, Waltham, USA) according to the manufacturer’s instructions. Polymorphism rs211105 was assessed by polymerase chain reaction - restriction fragment length polymorphism (PCR-RFLP) according to the method described by Shiotani et al. [21] with own modification. Primers for PCR reaction had the following sequence:

TPH1HAF forward primer: caaaagcagaataaagatgcaca and.

TPH1HAR reverse primer: acctacagggtgagggaagg.

The program in a thermocycler consisted of and initial denaturation at 94 °C for 3 min, a proper denaturation at 94 °C for 30 s, attaching the primers at a temperature of 61 °C for 30 s, synthesis at 72 °C for 30 s, and final synthesis at 72 °C for 5 min. The number of cycles was 40, after which cooling was performed at 4 °C. There was 25 µl of the mixture of DreamTaq™ Green Master Mix (Thermo Scientific, Waltham, USA), specific primers, the DNA matrix and molecularly pure water (Sigma-Aldrich, Saint Louise, USA). The yield and specificity of PCR products were evaluated after electrophoresis in 1.5 % agarose gel (Promega, Madison, USA) and stained with GelGreen (Biotium, Fremont, USA). Next, FastDigest® BsuRI (HaeIII) (Thermo Scientific, Waltham, USA) enzyme was added to the *TPH1* rs211105 PCR products and then digested according to manufacturer’s instruction. For genotyping, a 2.5 % agarose gel was used (Fig. 1). To confirm proper genotyping, 30 randomly chosen samples was genotyped one more time after proper genotyping. PCR-RFLP products were: TT (324 bp), GG (75, 249 bp), and GT (324, 249, 75 bp).

### Tryptophan hydroxylase 1 concentration

TPH1 concentration has been determined in plasma using Human Tryptophan 5-hydroxylase 1 ELISA kit according to the manufacturer’s instruction (Wuhan EIAab Science Co., China). The analysis was performed in duplicate at 37 °C with gentle shaking (250 rpm) in a microplate incubator (SkyLine ELMI Shaker DTS-4, Riga, Lithuania).

In brief, TPH1 content was measured in the following order: 100 µL of Samples, Blank, and Standards in range of concentration 0.312 – 20 ng/mL were added into microtiter strips and incubated for 2 h. After removal of the liquids, 100 µL of Detection reagent A working solution was added. Incubation was carried out for 1 h, after that the microplate was rinsed three times with Wash Buffer, and 100 µl of the Detection reagent B working solution was added. After a 1-hour incubation, rinsing the microplate with Wash Buff-er was performed as previously, and 100 µL of Substrate Solution was added to each well. After a 15-minute incubation, 50 µL of Stop Solution was pipetted to the microplate. The absorbance was measured at a wavelength of λ = 450 nm using an ELISA reader (BiogenetAsys UVM 340, Cambridge, UK).

### Serotonin concentration

The analysis was performed in duplicate using the Serotonin ELISA kit according to the manufacturer’s instruction (LDN, Labor Diagnostika NORD, Nordhorn, Germany), and described before by Cieślińska et al. [30]. All steps of the ELISA were carried out at RT (room temperature) with gentle shaking (250 rpm) in microplate incubator (SkyLine ELMI Shaker DTS-4, Riga, Lithuania). Samples were acylated before analysis as follows: 25 µL of serum, standards or controls was mixed with 500 µL of acylation buffer and 25 µL of acylation reagent provided with the kit. The mixtures were incubated for 15 min at RT. Serotonin content was measured as follows: a standard curve in concentration 10.2 – 2500 ng/mL, controls and serum samples were pipetted into serotonin microtiter strips. Then, 100 µl of the serotonin-specific antiserum preparation was added into all wells and incubation was carried out for 30 min. Plate was washed three times with Wash Buffer and 100 µl of the conjugate was added. After 15 min of incubation, 100 µL of substrate was pipetted. A 15-minute incubation was repeated and 100 µL of stop solution was added. The absorbance was measured at a wavelength of λ = 450 nm using an ELISA reader (BiogenetAsys UVM 340, Cambridge, UK).

### Statistical analysis

The frequency distribution of common risk factors for AP are presented as the mean. The genotype distribution among subjects was analysed for Hardy-Weinberg equilibrium (HWE) using the chi-square test, and genotype and SNP allele frequencies were compared in AP patients and control groups by Fisher’s test. Odds ratios (ORs) and 95 % confidence intervals (CIs) were calculated using logistic regression analysis and used to compare both allele frequencies in controls and AP patients, and allele frequencies between females and males. The risk of AP development was estimated via wild-type genotype and wild/mutant versus the mutant-type genotypes. Serotonin and tryptophan hydroxylase 1 concentration results were used to determine the distribution of variables, and are presented as a mean ± standard error. The mean values in Control and AP groups were compared using ANOVA and Student’s t-test. One-way analysis of variance was performed for multiple comparisons between groups, and Tukey’s honestly significant difference test was used to conduct post-hoc analysis. Spearman’s rank correlation coefficient analysis was used to estimate the relationship between analysed parameters. Statistical analysis was calculated using Statistica 13.1 (TIBCO Software Inc., Paolo Alto, CA, USA) and GraphPad Prism 6 software (GraphPad Software Inc., San Diego, CA, USA), with ≤ 0.01 P-value considered statistically significant.

## Results

### Polymorphism rs211105 in the TPH1 gene

At the rs211105 polymorphic site the frequency of alleles T and G were determined in healthy individuals and in those diagnosed with AP in our study population. Concentrations of serotonin and TPH were used to determine the normality of distribution (W > 0.90; p < 0.05 for the study and control group).

The three genotypes (TT, GT and GG) were identified in the whole study population (Control and AP). Of the total 198 participants, 108 had genotype TT, 79 had GT and 11 had GG. The observed genotype frequencies at rs211105 polymorphic site of TPH1 gene in Controls (χ2 = 0.73, p = 0.39) and AP patients (χ2 = 0.11, p = 0.74) conformed to the Hardy-Weinberg equilibrium. This suggests no unexpected population stratification and no sampling bias.

Table 2 shows the genotype distributions, allele frequencies and associations between genotype at the rs211105 polymorphic site and the associated AP incidence. We determined an association between the presence of the T allele at the position rs211105 (OR = 2.47, 95 % CI 0.94–6.50, p = 0.06) of the tryptophan hydroxylase 1 gene under conditions of a decreased AP incidence. We also noted that in AP group in comparison to Control group genotype GG at the position rs211105 is more frequent than GT (OR = 2.01, 95% CI 0.49–8.16, p = 0.32) and TT (OR = 2.67, 95% CI 0.67–10.59, p = 0.16).

**Table 2:** Genotype and allele frequencies of rs211105 in TPH1 gene polymorphism in studied groups and T-allele acute pancreatitis association

Genotype/Allele	AP n (%)	Control n (%)	OR (95 % CI) Control vs. AP	p-Value
TT	54 (50.5)	54 (59.3)	–	
GT	45 (42.1)	34 (37.4)	1.32 (0.74-2.37)	0.34
GG	8 (7.5)	3 (3.3)	2.67 (0.67-10.59)	0.16
G	61 (29)	40 (22)		
T	153 (71)	142 (78)		
Control vs. AP TT+ GT vs. GG			2.47 (0.94-6.50)	0.06

*CI* confidence interval, *OR* odds ratio, *AP* acute pancreatitis patients

### Tryptophan hydroxylase 1 concentration in serum

**Fig. 2:**
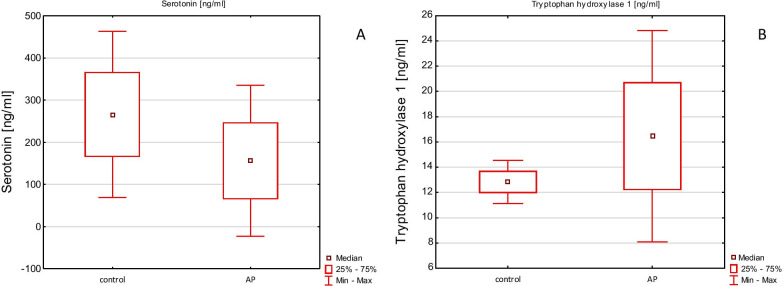
Serotonin (**A**) and tryptophan hydroxylase 1 (TPH) (**B**) concentrations in serum (ng/ml) in control and AP groups

**Fig. 3:**
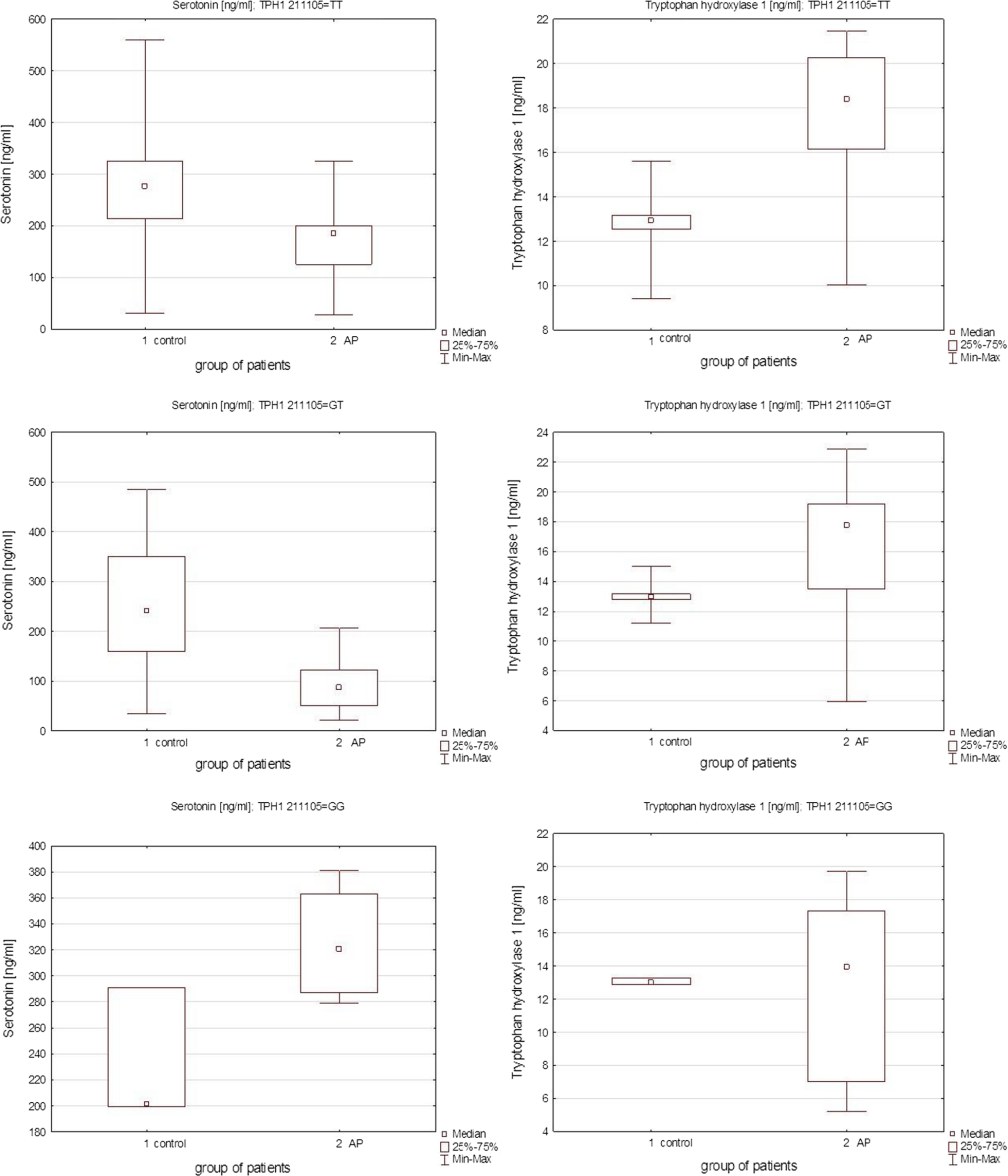
Serotonin and tryptophan hydroxylase 1 (TPH) concentrations in serum (ng/ml) in correlation to TPH rs 211105 TT, GT and GG genotype in control and AP group

The average TPH1 concentration in the Control group was 12.8 ng/ml (SDE = 0.09), and in the AP group 16.5 ng/ml (SDE = 0.41) with a statistically significant difference (p < 0.0001). Figure 2 presents the tryptophan hydroxylase 1 concentrations according to TPH1 rs211105 (T/G) genotype in both control and AP groups. The largest difference was found between the control and AP groups with the GG genotype (12.7 ng/ml, SDE = 1.97). Tryptophan hydroxylase 1 (TPH) concentrations in serum (ng/ml) in correlation to genotype are presented in Fig. 3.

**Fig. 4:**
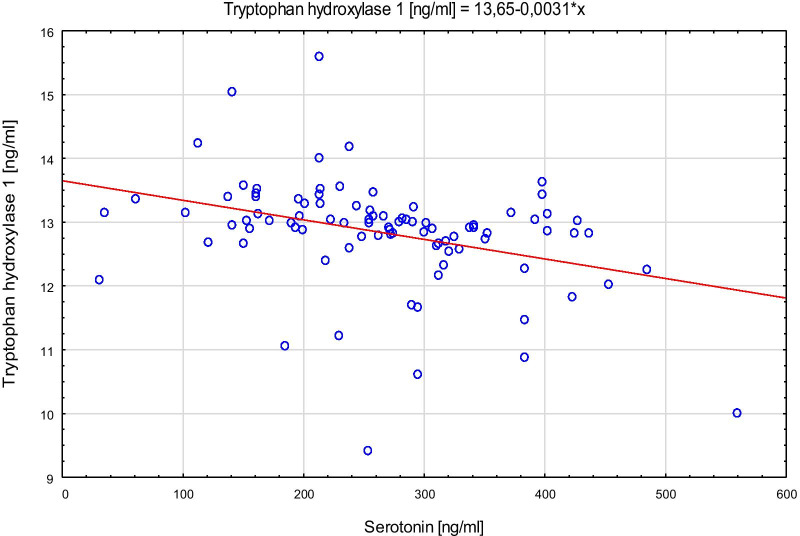
Scatter plot of tryptophan hydroxylase 1 [ng/ml] versus serotonin [ng/ml] in control group

**Fig. 5:**
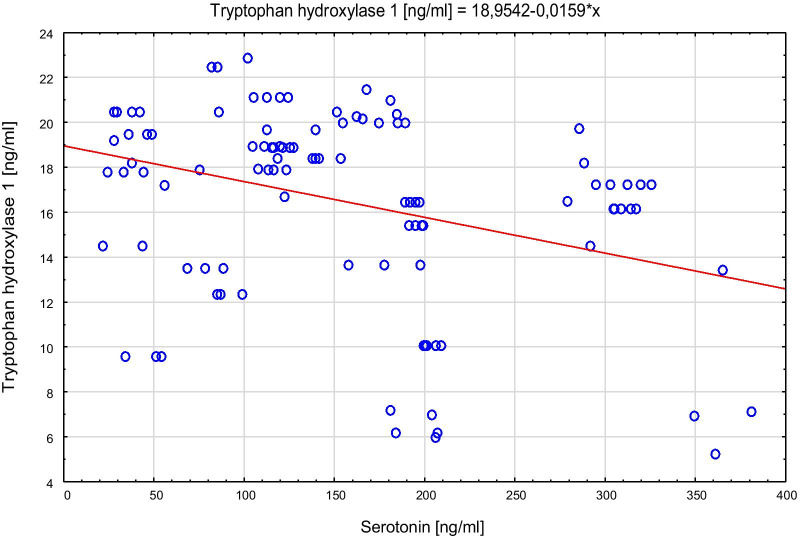
Scatter plot of tryptophan hydroxylase 1 [ng/ml] versus serotonin [ng/ml] in AP group

**Fig. 6:**
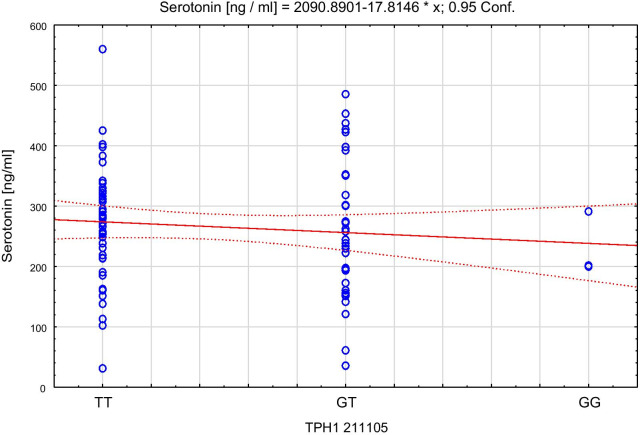
Scatter plot of serotonin [ng/ml] with genotype of rs 211105 in TPH1 gene in control group

**Fig. 7:**
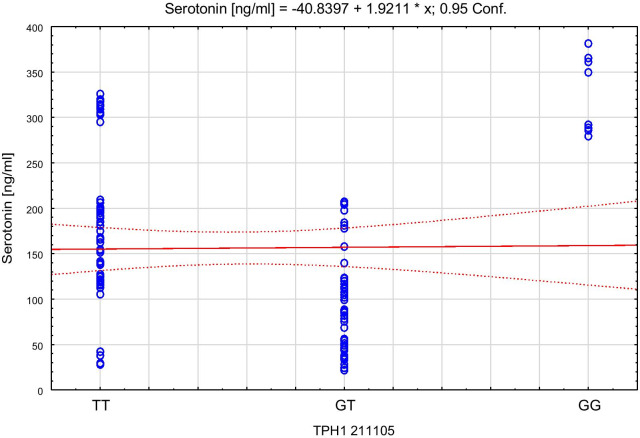
Scatter plot of serotonin [ng/ml] with genotype of rs 211105 in TPH1 gene in AP group

Spearman’s rank correlation coefficient with TPH, serotonin concentrations and the respective genotypes in both control and AP groups showed that among the control group lower levels of TPH correlated to higher serotonin level (TT: Rs = − 0.42; p = 0.002 and GT: Rs = − 0.46; p = 0.0066). Among AP group, Rs values were even higher (TT: Rs = − 0.75, p < 0.0001; GG: Rs = − 0.74; p = 0.04). The scatter plots of tryptophan hydroxylase 1 versus serotonin for the control group are presented in Fig. 4, and for AP group in Fig. 5. The scatter plots of serotonin with the different genotypes of rs 211,105 in TPH1 gene are shown in Fig. 6 for the control group, and in Fig. 7 for the AP group.

## Discussion

Pancreatic β cells are the main cell type regulating glucose and lipid homeostasis by the action of their insulin production which is controlled by nutrients (mainly glucose level), the nervous system, and the presence of other hormones, including melatonin, estrogen, leptin, growth hormone, and glucagon like peptide-1 [8]. There are several studies showing that pancreatic β cells are also capable of serotonin production, and contain the enzymes required for serotonin synthesis, including TPH1 and TPH2 [8, 31, 32].

Combined genetic, metabolic and environmental factors contribute to the development and reoccurrence of acute and chronic pancreatitis [33]. To the best of our knowledge, this is the first examination and association of TPH1 gene polymorphism rs211105 and serotonin and tryptophan hydroxylase concentrations in patients diagnosed with acute pancreatitis.

The present study involved 198 individuals: 91 Controls and 107 AP patients. The results of laboratory parameters determined prior to analysis are presented in Table 1. Higher levels than accepted reference points for bilirubin, ALT, and AST were determined in the AP patients (1.9 mg/dL, 155.7 IU/L and 155.2 IU/L, respectively). AP patients also had increased amylase activity, significantly higher lipase activity indicating pancreatic dysfunction. Their additional high p < 0.001 CRP level demonstrates an ongoing and chronic inflammation.

It has been known that serotonin has an important role in the development of experimental colitis pathogenesis and causes the secretion of proinflammatory mediators in the immune system. The regulation of 5-HT and 5-TH expressing cells is closely correlated with level of inflammation, which is characteristic in many diseases of the digestive system and various types of cancer [7, 34]. In energy metabolism, crucial roles are played by insulin, glucagon, and serotonin, whose concentration is regulated by the amount of glucose in the human body [31]. Here, we have shown a statistically significant correlation between the genotype of TPH gene and levels of serotonin.

The serotonin level in the control group was higher than in the AP group. The inverse correlation was related to the concentration of TPH, which was higher in the AP group compared to the control group. It is worth emphasizing that with a lower concentration of TPH in the control, a high concentration of serotonin is still maintained, while in the AP group - despite a high concentration of TPH, serotonin levels are lower (Fig. 2).

In the AP group, the TT genotype was linked to a higher TPH concentration and to the lowest serotonin levels (Rs = − 0.75, p < 0.0001) in comparison to the control group, where the TT and GT genotype subgroups had a less strong correlation between serotonin and TPH levels. Only in the AP group of patients with GG genotype, serotonin concentration is on average higher than in the control. It has been known that the intracellular content of serotonin correlates positively with insulin secretion, which is the main factor conditioning normal glycemia [12]. Thus, we anticipate that low level of serotonin in patients with acute pancreatitis could possibly result in disruption of the synthesis of insulin, which resulted in a pathological high and sustained glucose concentration. This mechanism is consistent with the glucose concentration of the participants, where the statistically higher glucose level was related to the AP group (p > 0.05) as presented in Table 1.

TPH is closely related to serotonin synthesis, what has been described by many researchers [7, 34]. The inhibition of serotonin production using a specific inhibitor of TPH1 decreases the severity of trinitrobenzene sulfonic acid-induced colitis in mice, indicating that the enzymatic regulation of HT-5 synthesis may influence on the development of improved therapeutic strategies in inflammatory disorders [35]. In our study, serotonin concentration was negatively correlated with the TPH1 concentration. Additionally, in AP patients with the TT genotype their higher TPH levels were negatively associated with serotonin concentration in comparison to controls. Thus, we showed that despite the high TPH1 concentration in the AP group, these patients had a lower serotonin concentration compared to control (Fig. 2). It is suggested that our results are in conflict with the widely accepted mechanism that the synthesis of serotonin from tryptophan is enzymatically regulated by tryptophan hydroxylase in a positive correlation. However, it should be noted that our research included the analysis of the concentration TPH 1, and not its activity. Presumably, a high concentration of enzyme is not always correlated with high catalytic activity, which we described in our previous studies on the role of dipeptidyl peptidase-4 (DPPIV; EC 3.4.14.5) in autism spectrum disorders [36].

Our current results determined the relationship between serotonin and TPH-1 levels with genetic factors, including the polymorphism rs211105 in the TPH1 gene in healthy and AP patients. Table 2 shows distribution of genotypes and alleles frequencies. Our data show that three genotypes (TT, GT and GG) could be identified in both groups, with the TT genotype being predominant among all 198 participants.

We have determined that genotype GG at the position rs211105 in AP group is more frequent than GT and TT in comparison to Control group. In this study, we have also found an association between the presence of the T allele at position rs211105 of the tryptophan hydroxylase 1 gene under conditions of a decreased AP incidence. Thus, the difference in both, TPH1 concentration and polymorphism rs211105 in TPH1 gene may indicate a putative role of this enzyme in the availability of serotonin in the human body and thereby a probable impact on the development of diseases associated with glucose metabolism. This issue is highlighted by Katsumata et al. [22], by showing that the group of patients with diarrhoea predominant irritable bowel syndrome showed a significant correlation between the TPH1 rs211105 T/T genotype and their lower scores for physical and mental health, and higher scores for indigestion and diarrhoea. The frequencies of the TT/GT/GG genotypes were: 48/13/1 (0.88 for T and 0.12 for G alleles), and 46/18/0 (0.86 for T and 0.14 for G alleles) for diarrhoea-predominant irritable bowel syndrome patients and controls, respectively. These presented frequency results are also similar to Gizatullin et al. [25], who showed a rs211105 frequency of the T allele of 0.75 (G 0.25) in the control group and 0.78 in major depression patients (G 0.22), and also Andreou et al. [26] in healthy volunteers – with a 0.23 frequency for the G allele. An important aspect is the influence of alcohol consumption on the development of acute pancreatitis. It is indicated that this factor is responsible for approximately 17-40 % of the causes of AP [37, 38]. It was also shown that one of the known SNPs in the TPH1 gene (rs1800532) is a potential biomarker for bipolar disorder and alcohol-dependence risk in the Caucasian population [39]. In our study, we did not take into account the relationship between the presence of the rs211105 polymorphism in the TPH1 gene and its relationship with alcoholism, because of conflicting data in the literature on selected polymorphisms in the TPH1 gene and its association with alcoholism [40]. In our previous research (unpublished data), we also did not find a relationship between the occurrence of alcoholism and the rs211105 polymorphism, however, due to the small study group (30 individuals), these data were not included in the current publication, and were used for additional analysis when selecting the control group.

We have made every effort to obtain fair and unbiased research. Nevertheless, we are aware of some limitations that may have occurred in the planning of this study. The following aspects should be mentioned: (I) lack of information about alcohol consumption by individuals in the control group, (II) limited number of participants in the study, (III) no follow-up monitoring of the health of participants after leaving the hospital, (IV) bias towards males in alcohol consumption in both groups. Further studies should take these considerations into account.

## Conclusions

The aetiology and the course of AP are still insufficiently examined. Here, a new insight on the potential role of a serotonin-related genetic factor in pancreatitis development is described. Factors affecting serotonin synthesis may be an interesting and important consideration in acute pancreatitis. The present research focuses on an important disease from the point of epidemiology, which requires novel and fast diagnostics that is extremely important due to the prevention or alleviation of symptoms of this devastating disease. Undoubtedly, early diagnostics of acute pancreatitis combined with minimizing the adverse effects of malfunctioning pathways by choosing the right diet is a significant challenge for nutrigenomics. Therefore, the research carried out on the correlation between polymorphisms in genes and their influence on the action of TPH1 enzyme on the body, blend into the contemporary nutrigenomics trend and emphasize the role of biochemical individuality in the creation of the basis of nutrition and personalized medicine.

## Data Availability

The datasets used and/or analysed during the current study are available from the corresponding author (Anna Cieślińska) on reasonable request.
